# The Role of European Equestrian Institutions in Training Professionals: Outcomes from a Workshop on Horse Welfare in Equestrian Education

**DOI:** 10.3390/ani15020183

**Published:** 2025-01-11

**Authors:** Gabriella Torell Palmquist, Nina Känsälä Alveheim, François Huot-Marchand, Lisa Ashton, Victoria Lewis

**Affiliations:** 1 Department of Educational Studies, Karlstad University, 651 88 Karlstad, Sweden; 2 The Swedish National Equestrian School of Excellence, 734 94 Strömsholm, Sweden; 3 Department of Animal Biosciences, Box 7023, Swedish University of Agricultural Sciences, 750 07 Uppsala, Sweden; nina@djursholmsridskola.se; 4 L’Institut Français du Cheval et de L’équitation, 49400 Saumur, France; francois.huot-marchand@ifce.fr; 5 Equestrian Performance Research Centre, Hartpury University, Gloucester GL19 3BE, UK; lisa.ashton@hartpury.ac.uk (L.A.); victoria.lewis@hartpury.ac.uk (V.L.)

**Keywords:** equestrian education, ethical training, equine welfare, sustainable equestrian, social license to operate

## Abstract

This paper highlights and summarizes the experiences and perceptions of European equestrian educational experts from a workshop held at the French National Riding School in Saumur. The workshop, which brought together fifty leading experts, aimed to address challenges in equestrian education, identify common ground, and share best practices. Using ‘The World Café’ method, the participants were divided into groups to discuss four key topics: the relationship between horses and humans, diversity of perspectives versus common ground, training philosophy, and social acceptability. A follow-up online focus group was conducted six months later, and the discussions were transcribed and analyzed using qualitative content analysis. The results revealed the need for a universal cultural shift towards an evidence-based approach to riding and training horses, with equine welfare at its core. Key challenges, such as anthropomorphism, varying standards across disciplines and countries, and the evolving horse–human relationship, pose risks to the social license to operate. The findings suggest that educational institutions, with their shared values, are well-positioned to lead this change, although further research is needed to address pedagogical challenges. The study also emphasizes the importance of communicating ’good news stories’ to promote evidence-based practices in training and competition.

## Simple Summary

This paper summarizes the experiences and perceptions of European equestrian educational experts from a workshop at the French National Riding School in Saumur. The workshop addressed key challenges in equestrian education, focusing on topics such as the horse–human relationship, diversity of perspectives, training philosophy, and social acceptability. The findings highlight the need for a cultural shift towards an evidence-based approach to training, with equine welfare at its core. Key challenges, including anthropomorphism and varying standards, threaten the social license to operate. The study suggests that educational institutions can lead this change, with further research needed on pedagogical challenges.

## 1. Introduction

Horses have been domesticated and trained by humans for use in agriculture, transportation, the military, and, more recently, sport and leisure. Equitation, the practices and philosophies of riding and training horses, can be traced back to ancient Greece (Xenophon), where the fundamental principle of training horses, as with most other animals, involves shaping behavioral responses [1]. Horses satisfy their species-specific needs (telos—the unique characteristics of their anatomy, physiology, ethology, and cognition) by oscillating between resolving negative experiences, such as hunger, thirst, pain, discomfort, and predator avoidance, and exploring opportunities for positive experiences, including curiosity, play, and sexual instincts [2]. Training taps into these species-specific behaviors by reinforcing desired responses [3,4].

Equine welfare refers to the physical and mental state of the horse, as well as the degree to which their natural instincts and inherent needs (telos) are fulfilled [2]. Good welfare and training are not simply about avoiding negative welfare impacts but also about promoting positive welfare impacts [5]. The concept of ‘A Good Life for Horses’ (from the Interim Report to the FEI Sports Horse Forum, April 2023, by the Equine Ethics and Wellbeing Commission) is constantly evolving as the evidence base provided by welfare science develops [5]. The EEWC report highlights that the concepts of ethics and equine welfare are often related. That is, according to the report, how we determine whether something is ethical depends on factors such as personal ethics, professional ethics, and ethical perspectives like utilitarianism or deontology. Ethics refers to the philosophical study of moral concepts, theories of right and wrong, good and bad, and systems or codes of moral rules, principles, and values [6].

Equine welfare concerns how we best manage horses to optimize their well-being, assuming we believe it is ethical to use horses in sport [5]. Although ‘ethics’ and ‘welfare’ are separate concepts, they are linked, as our ethical choices (regarding what is ‘acceptable’) are strongly shaped by our awareness of the welfare consequences [5]. The goal should be to ensure that horses have a “Good Life”, where their experience over time is predominantly positive [5]. Prioritizing equine welfare is essential, both as an ethical duty to the horse and for maintaining a productive working relationship.

A social license (social license to operate) depends on the perceived legitimacy, credibility, and trustworthiness of equestrians, linked to society’s moral values [5]. Ethical frameworks can help maintain the social license to use horses in sport and enable those within equestrianism to critically assess existing and proposed practices, as well as make welfare improvements where needed [7]. The decline of several animal-use activities highlights that the sustainability of equestrian sports depends on addressing ethical considerations and equine welfare [8]. Equine welfare is best ensured by addressing concerns from both within and outside the equestrian community. In recent years, research related to equine welfare in equestrian sport has also demonstrated that horses’ natural needs are not satisfied (see for example: Douglas et al., 2022; Holmes and Brown 2022; Luke et al., 2023; Kelly et al., 2021; Mellor et al., 2020; Pearson et al., 2023; Wolframm et al., 2023) [2,8,9,10,11,12,13].

Videos of horse abuse have brought into question horse trainers’ choices, with a spotlight on in-hand and under-saddle interactions based on force (relentless pressure), punishment, or the use of the horse as a commodity (winning over well-being) [14,15,16]. This has raised critical concerns about ethical considerations and welfare aspects of horse training and competitions. This has challenged equestrian governing bodies and stakeholders to acknowledge the need for public acceptance of using horses in sport and to explore strategies for securing public approval, ensuring the future of equestrianism.

Equestrian organizations have for a long time been engaged with horse welfare issues, and, recently, their efforts have intensified. The European Equestrian Federation (EEF) states that it is time for a paradigm shift, urging national equestrian federations to lead in educating trainers, riders, officials, and other stakeholders on ethical training methods [17]. The independent organization World Horse Welfare, which advises the FEI and the International Horse Sports Confederation, emphasizes the need for research and education to significantly impact welfare in horse sports [18]. A leading strategic pathway is education [5], where established equestrian education institutions offering programs in riding, driving, and equine knowledge have a responsibility to update their curricula with evidence-based information. This should be combined with professionals engaging with, understanding, and communicating scientific insights to promote and support both the mental and physical well-being of the horse [2]. Such efforts are crucial for gaining public support for the use of horses in sport.

The aim of this paper is to highlight and summarize European equestrian educational experts’ experiences and perceptions from a workshop that focused on educating professionals and future professionals. The workshop was held at the French National Riding School in Saumur. It was the first of its kind to bring together leading European experts in equitation education. With this paper, we would like to address challenges, identify common ground, share best practices, and provide suggestions for the future development of equestrian education. These suggestions aim to consider both equine welfare and societal approval. An objective of this paper was to synthesize diverse expert opinions into a unified viewpoint and provide a baseline of expertise and opinions to support future research.

## 2. Materials and Methods

### 2.1. Participants

Fifty experts from 14 European countries were selected and invited by the Institut Français du Cheval et de l’Équitation (IFCE) to a three-day workshop at the French National Riding School in Saumur. Most of the experts were members of the Equestrian Educational Network (EEN) and had expertise and experience in equestrianism as coach educators, international riders, members of National Governing Bodies, academics, and leading equestrian specialists. They attended as representatives of the leading equestrian educational institutions, and several of them were also members of the International Group for Equestrian Qualifications and the Fédération Equestre Internationale.

### 2.2. Measure

To focus discussions and ensure meaningful outcomes from the workshop, the invited participants attended four expert-led presentations on the first day. The speakers were Sophie BARREAU (Teacher at Blondeau school of young horses and Research member of INRAE’s Animal’s Lab— Nouzilly, France), Dr Stephane MONTAVON (Vice-President of EQUI-SCOPE—formerly COFICHEV—in Hauteville, Switzerland), Markus SCHARMANN (Head of National Training Centre in Warendorf—Warendorf, Germany), and Laurence BLONDEL (Head of the individual high-level athletes support system at the INSEP—Paris, France). The presentations were intended to provide perspectives related to the purpose of the workshop. Four topics were collectively highlighted by the participants for discussion and summarization. These were as follows:

1. Understanding/explaining the relation between horses and humans.
2. Diversity of perspectives vs. common ground—shared values.
3. Training philosophy.
4. Dealing with social acceptability.

The participants were randomly divided into four groups, with four volunteers (among the participants) moderators for each topic. Focus groups utilizing the ‘World Café’ method were conducted. The World Café [19] is an engagement process designed to take place in a café-like setting—either in an actual café or in a room arranged to resemble one as closely as possible, with participants seated around small tables. The purpose of this setup is to create an environment that fosters meaningful conversation, allowing participants to discuss topics that matter to them. The method operates on the assumption that people inherently possess the wisdom and creativity to address even the most challenging issues. It is founded on two key principles:

1. humans want to talk together about things that matter to them;
2. and, if they do, they can create collective power, for example, to drive change.

### 2.3. Protocol

Within ‘The World Café’, each group spent 45 min at each table, discussing the same topics (described earlier in the text) for that table before moving on to the next round. Each round was based on one of the topics related to the overall purpose of the seminar.

The moderators were provided with pens and encouraged to draw and document the conversations on paper to capture ideas as they naturally emerged. Each moderator stayed at their table and summarized the previous discussion for newly arrived participants. By rotating participants around the room, the conversations at each table were enriched with ideas from other groups, culminating in a collective summary. The moderators’ role was to ensure that everyone’s contributions were valued and that participants actively listened and explored insights together. They were also responsible for managing the time effectively.

After the sessions, all participants were invited to a round-table plenary where the moderators collated all the information gathered from each round and presented the information to the whole group. A further focus group lasting 60 min was carried out, and overall findings and conclusions were agreed and reordered on a white board by the lead moderator.

### 2.4. Six Month Post-Workshop Focus Group

A post-workshop focus group was carried out online on Zoom, six months after the workshop. Part of the authors (VL and GTP) of this paper gave the participants the opportunity to reflect on the workshop and to discuss any topics that have developed in the equine industry since the workshop. The workshop was recorded and transcribed.

### 2.5. Data Analysis

The transcriptions from focus groups (World Café) and post-focus group discussions were analyzed using qualitative content analysis, following the method of Graneheim and Lundman [20]. The analysis focused on condensing and interpreting meanings. First, the transcripts were read in full, and then key sentences and phrases were identified as meaning units and coded. These codes were organized into lower-order themes and challenges to reflect their content. Finally, the codes were filtered and grouped by topic to summarize the participants’ discussions and conclusions.

## 3. Results

The result is presented descriptively and reports on the collective thoughts and experiences of the participants. Table 1 illustrates the analysis of the focus groups.

### 3.1. Topic 1: Horse–Human Interaction

#### 3.1.1. Past and Present

The discussion among the participants focused on how the role of horses has changed over time throughout Western society from being essential for agricultural work and transportation to being used primarily for leisure and sport.

The ways in which we use and train horses have also evolved. In the past, horses were vital to the functioning of society. Horses were a necessity, and their value was measured by their ability to perform specific tasks efficiently.

(Note from round table: Category 1. Horse human interaction)

Participants agreed the relationship between humans and horses has also changed.

In the past, horses were viewed primarily as tools, and their welfare was often overlooked. Nowadays there is a greater emphasis on the ethical treatment of horses, with many people seeing them as partners and companions. It is important to pay attention to the history and to understand equestrian cultures, especially as equestrian educators are part of creating the future through their students.

(Note from round table: Category 1. Horse human interaction)

#### 3.1.2. Communication and Interaction

The relationship between horses and humans involves communication and interaction.

As a rider, it is important to consider how to interact and communicate with the horse. This is a crucial part of equestrian education.

(Note from round table: Category 1. Horse human interaction)

The participants believe that it is important to be clear when conveying to the students that horses do not think like humans, highlighting the danger of anthropomorphism on equine welfare.

They must learn and understand how to interpret the horse’s body language and read, listen, and observe the horse. To do this, it is important to be authentic and distinct in the relationship with the horse so that the horse understands what we want it to do. This requires a clear educational training system and promoting a respectful attitude.

(Note from round table: Category 1. Horse human interaction)

Participants felt that, to protect equine welfare, it is important to select individual horses carefully. Matching horses with the most suitable rider in terms of size, ability, and temperament is essential.

#### 3.1.3. Research-Based Knowledge

The results showed a need for greater knowledge about the relationship, communication, and interaction between horse and human, as well as a recognition that scientific knowledge must be more widely disseminated within the equestrian community.

There is a lot of research-based knowledge, but much of it does not reach the large mass of horse people. The reasons for this may vary. It may also be that the knowledge exists, but it is not used to a sufficient extent or in the correct way. It may also require a new approach, time, and effort to understand what needs to change.

(Note from round table: Category 1. Horse human interaction)

It was agreed that equestrian education organizations are important forums for objective and critical discussions on evidence-based research. Teaching in this area has evolved over time and continues to develop as new research emerges. Graduates can play a key role in sharing this knowledge, fostering curiosity among riders about improving their communication and interaction with horses. The participants also agreed that it is important to explain to students to adapt training practice to the individual horse and not to go too fast regarding educating horses. Therefore, educators must explain the need to be predictable and precise. There is plenty of traditional knowledge that is worth trading, but it is also important to absorb new knowledge based on research and from a perspective of horse welfare.

### 3.2. Topic 2: Shared Values

#### 3.2.1. Countries

As the workshop included representatives from various countries, different perspectives between countries were discussed and compared. The systems for riding instruction and horse education have many similarities. For riding instructors/coaches, there are qualification systems within the countries, and these are linked to the IGEQ’s international level system. Differences were highlighted, particularly regarding various rules and regulations, such as differences in young horse classes and qualification systems. Several of the represented countries had licensing systems where riders are registered to compete, but this did not apply to all competitions. In Sweden, there is also a green card system at the lowest level, as one of the participants mentioned. Generally, anyone can buy a horse in the represented countries, which poses issues concerning knowledge. Horse management varies not only between countries but also within countries. The EU has some legislation regarding animal husbandry, but the different countries also have their own more or less extensive regulations. The participants acknowledged that all these factors make regulating high welfare standards across countries difficult.

Some participants mentioned that the long-term future of equestrianism also relies on participation rates for financial sustainability. The participants expressed that a common challenge for riding schools is retaining young members, as many often stop participating when they reach adulthood. According to the participants, riding schools exist in all of their countries, with most students being girls from middle-class white families. Participants from France and Sweden mentioned that riding schools in their countries offer subsidized activities for younger children and run projects aimed at increasing participation levels. Based on the participants’ accounts, a challenge for inclusion and diversification can be observed. Indeed, most participants stated that their student population primarily consists of white, middle-class females. Examples were also provided of some institutions implementing targeted outreach programs or subsidies to attract male students, individuals from lower-income backgrounds, and ethnic minorities. From a broader perspective, equestrian cultures around the world differ significantly, leading to diverse views on the role and use of horses. The participants agreed that global issues, such as inflation, war, climate change, and the social license to operate, affect equestrian sports and leisure.

The future cannot be predicted, but it is important for equestrian educators to make students aware that changes are happening.

(Note from round table: Category 2. Shared Values)

#### 3.2.2. Disciplines

Equestrian sports consist of a variety of disciplines that are found to varying degrees in different countries. The educators discussed the fact that some disciplines have greater or lesser popularity. There are attitudes and misunderstandings between groups and disciplines. Different ethical differences are based on tradition and history and/or economics. Within competitive sports, there are also cultural differences in terms of rules. Traditions and history can vary in strength depending on their roots in each country, all of which could impact welfare standards. The participants have identified that the traditional disciplines found within the Olympic events have lost some of their appeal, and some of them experienced that more people are turning to Natural Horsemanship-inspired variations.

Social media and influencers on platforms like YouTube showcase different forms of practice, which may not always prioritize safety/welfare. There is a lack of knowledge about where these followers acquire their knowledge and understanding of companion animals.

(Note from round table: Category 2. Shared Values)

The participants emphasized the importance of drawing students’ attention to this form of influence and approaching it critically from both a safety and horse welfare perspective. At the same time, they also saw this as an opportunity to leverage these platforms for positive change.

### 3.3. Topic 3: Training Philosophy

#### 3.3.1. Ethical Issues

One of the main discussion points raised was ethical issues in relation to educating and training horses for sport and leisure. The participants agreed that training and performance have been a subject of debate for a long time. The participants discussed and mapped out common issues. For example, young horse competitions. Several European equestrian federations have competition systems where young horses can be tested against an educational standard level. The systems have developed and changed over time, but there are still adjustments that can be made to strengthen the training and preparation of young horses. Another issue is high-level performance; there is a growing concern over the ethical treatment of horses during training and competition at this level. Achieving high-level performance without harming the horse is possible through ethical training methods that prioritize the horse’s well-being, the participants agreed.

The key to successful horse training is to understand the horse’s natural behavior and adjust the training accordingly.

(Note from round table: Category 3. Training philosophy)

Standpoints raised that training should be gradual, and horses should be given ample time to rest and recover between training sessions. The discussions also produce more questions concerning ethical issues of training philosophy. Questions concerning the right amount of load the horse can accept were raised, as well as whether we have reached the limit (physical, psychological, cognitive, and emotional).

#### 3.3.2. Norms and Traditions

There are various cultures in which horses are trained and educated, many of which are based on norms that have been passed down over time within their respective cultures. Today, the participants claimed, an important aspect for riders and trainers to consider is to make visible the prevailing norms and critically examine them based on aspects such as horse welfare and ethics. For example, in the context of discussions regarding young horse classes, norms can be scrutinized to determine whether the standards set to pass tests are realistic and how riders and horse owners should respond if a horse does not meet the norm-based requirements. It is valuable to clarify the importance of individual adaptation based on the horse’s capabilities and level of development.

#### 3.3.3. Transparency

The participants felt that there is a need to explore how riders can work and train horses in an ethical way. There is also a need to examine the risks associated with horse training and performance and the measures that can be taken to minimize them.

Horses have their own set of needs and behaviors, and it is crucial that their training is based on a deep understanding of their physical and emotional well-being. The use of force and fear-based training techniques can cause severe physical and psychological harm to horses, which can result in long-term damage to their mental and physical health.

(Note from round table: Category 3. Training philosophy)

The participants suggested a solution would be creating a more transparent training philosophy with a more specific focus on horse welfare. To achieve that, a common goal among riders and coaches is needed where the horse’s well-being is put at the center. One of the groups highlighted how trainers and riders can use zones as a concept in horse education.

The first zone is described as the Resting zone, where the horse is not working but resting, while the Comfort zone is described as the zone where the horse is usually worked. When something new is introduced, the horse enters the Training zone. There, the horse has the opportunity to familiarize itself with new elements and/or environment. There is another zone that one should avoid the horse from entering, and that is the Panic zone. It is primarily related to the horse’s tendency to flee and causes discomfort for the horse.

(Note from round table: Category 3. Training philosophy)

The participants agreed that it is important to reflect on how to handle and train horses and for what purpose. “*The path to the goal is at least as important as the result itself*”. It was also acknowledged by the participants that there is still a need for tools to objectively monitor the workload. The discussion among all groups concluded that there is a need for clarification and transparency in how horses are trained for sports and recreation. It is important to not blindly follow norms but to question and reflect on one’s own approach.

Horses are individuals and differ from one another; hence, there should be flexibility and understanding in the approach to training and education of horses. Sometimes, it is necessary to deviate from the system to adapt and think outside the box, which may require compromising one’s principles.

(Note from round table: Category 3. Training philosophy)

In conclusion, working and training horses in an ethical way is possible through careful consideration and responsible action. It is essential to prioritize the welfare of the horse. Achieving high-level performance without harming the horse requires a deep understanding of the horse’s natural behavior and adjusting the training accordingly. Horse training and performance come with inherent risks, but these risks can be minimized through an appropriate and adapted training plan and time for recovery. By prioritizing the horse’s well-being, trainers and riders can achieve high-level performance while treating their horses with the respect and care they deserve.

### 3.4. Topic 4: Social Acceptability

#### 3.4.1. Communication

The participants concluded that communication is central; it is important to explain our interactions with horses. There are a lot of people in the world with no contact with horses, and therefore we need to include them. The horse industry needs to explain for lay people what we do with horses and why, and also explain about the horse’s natural needs.

The wording “using a horse”, should be changed to “in partnership”, as our language is powerful. We need transparency and courage to speak up and challenge if something in training or handling, for example, is abusive. There is a need in the horse world to be honest with each other, as this is a critical time for our future.

(Note from round table: Category 4. Social acceptability)

A common theme was that communication needs to be science-based and accessible with a clear summary.

This is needed for both lay people as well as people within the horse industry wanting to learn more about horses. Communication is the key word, and there is a need for evidence-based knowledge. Comment and explain interactions and spread the good picture.

(Note from round table: Category 4. Social acceptability)

The participants felt that the horse industry emphasizes partnership and equine welfare. Self-regulation within the industry involves responsiveness to societal concerns, prompting practitioners to intervene in questionable situations. They stated they play a crucial role in equipping students with an understanding of the diversity of ethical frameworks and intervention skills. While honoring tradition, the industry is faced with an opportunity to renovate and showcase the ability of professional educators to listen and integrate both societal feedback and evidence-based knowledge, shaping change for the future sustainability of equestrianism. Recommendations for improvement include establishing a global platform for discussion, enhancing education with a focus on equine perspectives, and reassessing norms to align with the principles of equine-centric horsemanship. Ensuring sustainability requires renovation, updating the curriculum for the future with innovative teaching and learning practices in the latest evidence-based knowledge.

The use of internet and social media makes it easier to get access to knowledge; however, this knowledge needs to be complete and correct, but much of the information available online is not.

(Note from round table: Category 4. Social acceptability)

According to the participants, this should be an opportunity to spread correct knowledge.

There is a need to organize our storytelling. The horse industry itself must be able to show what equestrianism is in a positive manner that showcases ethical training and high levels of equine welfare.

(Note from round table: Category 4. Social acceptability)

#### 3.4.2. Introspection

The participants state the need for a common voice but also to take self-responsibility and that self-reflective practice was important.

In order to protect the ‘SLO’, we must take personal responsibility for our own actions and challenge others when we see poor practices. Individuals must also reflect on their own practices—a skill that needs to be taught and developed

(Note from round table: Category 4. Social acceptability)

The participants agreed on the saying that the

Horse is the mirror of yourself. You don’t ask the question; the horse gives the answer. You see the horse; you see the man—it is the mirror.

(Note from round table: Category 4. Social acceptability)

Horses are important for social life; they help people to grow. Horses can give a good balance in the digital world of today. Children and young people can learn life lessons from horses, which is of benefit for the wider society. The equine industry urgently needs to explain the interactions between humans and horses to further social acceptance.

(Note from round table: Category 4. Social acceptability)

### 3.5. Follow-Up Focus Group

A follow-up online focus group was held with EEN members who also participated in the previous seminar in Saumur. During this workshop, the participants had the opportunity to further develop their reasoning and reflect on anything they had thought about or noticed since the seminar. The workshop began with the participants answering the question of how horse welfare is included in the school’s syllabus. The responses from the members indicated that horse welfare is included in several ways and across different subjects.

I would say that in every day and in every situation, we are talking what about the horse, the behavior of the horse, physiology, what about your ride and what’s going on and everything somehow… and every topic somehow horse welfare is touch.

(Interview Follow-up focus group)

There was a broad consensus on the importance of schools adopting a systematic ethical approach to training the horses. When the participants were asked to characterize what this training system looked like and how it operated, it became clear that it was not always explicitly outlined in the syllabus. Instead, it was integrated into the training system and had a holistic approach. Therefore, it became clear that it was important for students to understand that the approach is individualized for each horse; sometimes it takes longer, sometimes it takes less time. Some of the participants gave examples of how horse welfare was included, both in theoretical and practical aspects. They also highlighted the importance of students learning to read and interpret behavioral signals from the horses. The participants mentioned that they follow a systematic ethical approach to training their horses, but they find it challenging to provide a clear and concise description of the specific training system they use. Several of the schools verify that they have a developed educational system, but it is the individual horse’s mental and physical condition that determines the pace at which the horse follows all the steps. Having a policy document for horse welfare and continually engaging students in discussions about it seems significant. Also, implementing an ethical approach as a strategic plan, wherein all riders at the school are encouraged to be role models for horse welfare and understand the collective responsibility in maintaining a social license, appears crucial. A challenge, however, is to educate students with an equine-centric perspective, specifically identifying negative experiences for horses, attempting to resolve these negative experiences, and offering opportunities for positive experiences if the use of horses in sport is to be sustained. This is perceived as difficult.

I think we have a lot of knowledge and a lot of skills and lot of competence as we teach but the difficulty is to create horsemen, horsewomen with the right personality and the right attitude which is needed to assure horse welfare and sustainability in a sustainable way.

(Interview Follow-up focus group)

It is a challenge to meet the needs of educating students and meet horses where they are, the participants stated. Mistakes are how we know learning is happening. The participants emphasized that creating a psychologically safe environment, where students are encouraged to learn from their mistakes, is essential. Educators should guide and support them through these learning moments, while effectively applying modern learning theories. The proper use of pressure and release helps students improve their timing, consistency, and confidence in handling and training horses. The participants also highlighted the importance of giving students time to reflect on their goals, learn about themselves, and understand how they respond to different horses’ genetics, learning history, and current environment in various situations, while evaluating their impact on horse welfare outcomes. Thus, there is no preset system or strategic plan for instilling an ethical mindset in students. Instead, it is vital to address ethical issues as they arise within the group and discuss the importance of horse welfare and what it entails. Some of the participants emphasize the importance of understanding cultural differences between different countries. They believed it is important to recognize cultural diversity and discuss horse welfare together. There should be the possibility to discuss all the issues that arise and together innovate to renovate the state of equine education today. Therefore, there is a need to create psychological safety, where everyone feels safe to express themselves and engage in open conversation, and where dissent is welcomed and listened to, with the opportunity to rethink ideas or strongly held opinions. Having spaces where active listening is experienced facilitates diversity of thought and innovation potential. The participants viewed EEN as a valuable forum for these discussions, where challenges could be addressed, and perspectives and support could be obtained from the representatives of the schools. Consensus on these issues was also seen as important, especially when it comes to educating a diverse generation of riding instructors.

Since the seminar in Saumur, there have been some incidents during the past year where several cases of horse abuse in equestrian sport have been uncovered. The participants suggest that these incidents have prompted further discussions about horse welfare (the state of the individual, both physically and mentally) in the schools. Additionally, the seminar itself has led participants to reflect even more on the subject in relation to the content of the educational programs. In some cases, specific efforts have also been made within the framework of the topic.

…so we organize now every year with our students something about a clinic… there we have some veterinarian that talk about horse welfare and how to organize older school horses and that they feel good in every situation…how we can give them a little bit longer time to warm up… and so… and it was a good step in right direction but we have to go on with it.

(Interview Follow-up focus group)

There was a strong consensus from the group that such workshops and seminars should continue on a regular basis to discuss issues, share good practice. Collaborative projects to capture ‘good practice’ develop action research and address challenges collectively.

## 4. Discussion

The insights gained from the experts’ shared experiences and perceptions highlight several key challenges, identify common ground, and present best practices within European equestrian education. These findings provide a valuable foundation for further reflection and discussion. In this section, we will discuss these results, focusing on presenting suggestions that consider both equine welfare and societal approval to shape the future development of equestrian education. By linking these findings to previous research and theories, our aim is to synthesize the participants’ opinions into a unified viewpoint and establish a basis for future studies.

All participants had a common voice of concern for the current state of the equestrian world. Recent horse abuse such as the cases in Denmark [14], USA [15], and UK [16] have heightened this concern. There was a strong acknowledgement that the SLO is under severe threat, not only the use of horses in sport but also riding horses for human leisure and enjoyment.

### 4.1. Evidenced-Based Practice Supported by Current Equitation Science Research

The participants self-reported that the leading equestrian education institutions use current research to support the use of a systematic approach to training and riding horses. It was agreed that the application of scientific theory within practice is essential to the sustainability of the industry and imperative to protect the SLO. They felt strongly that educating the next generation of equestrian professionals must use evidence-based knowledge to achieve this.

The first step is the use of a scientific conceptualization of horse welfare. Knowledge has traditionally focused on the biological functioning of animals, where the provision of shelter, nutrition, and healthcare is equated with good animal welfare [21], and the idea of the three Fs (Friends, Forage, Freedom) is regularly used within the equestrian field [22]. However, there is much more to consider and learn when it comes to equine welfare, such as the current Five Domains model [2]. This incorporates nutrition, environment, physical health, and behavioral interactions (including interactions with both humans and non-human animals), which in turn contribute to the fifth domains: the animal’s mental state or affect.

Teaching animal science requires a regard for affective state or emotions (ISES principle 4), identifying the arousal level (high, neutral low), coupled with assessing when an experience for the horse is positive or negative (valence) to provide an accessible and immediate assessment of the emotional state of the horse [23]. Teaching the assessment of affect in educational institutions could help shift societal attitudes towards animal sentience, potentially ensuring the future sustainability of horse use in sport. However, while there is evidence of negative affect and injuries related to horse riding, further research is needed to explore potential signs of positive affect. In the meantime, it is crucial to base equestrian education on the existing evidence and be transparent about its limitations.

### 4.2. Safety of Horse and Rider

Within the systematic ethical training systems used by educators, the importance of ensuring the safety of both horse and rider is stressed throughout their interaction, including riding and competition (ISES 1st Principle). Riding is considered one of the most dangerous sports, with high risks of serious injuries [24,25,26]. For example, in Sweden, there were 1756 injury cases per year, and the medical cost for each injury was EUR 1800, or EUR 3 million annually [26]. These are also horse welfare concerns that threaten the SLO. Educators need to provide an environment that ensures the safety of both horse and rider by conducting risk assessments, ensuring the correct use of Personal Protective Equipment (PPE), ensuring riders are suitably mounted, considering the horse’s affective state, and using correct training techniques. These considerations should be incorporated within the syllabus.

### 4.3. Evidence Based Training Principles

For the participants’ educational training system to extensively utilize research, the following evidence-based principles must be applied: The First Training Principles [27] provide a blueprint for educational institutions to clearly demonstrate that these principles are integrated into their syllabus and daily horse training practices. Principles 5, 6, and 7 focus on communicating learning theory, including habituation, operant, and classical conditioning. To fully understand and apply these principles, educators must first recognize each mode of learning, then teach, assess, and improve students’ application, i.e., develop students’ operant contingency over time. To safeguard and future-proof themselves, educational institutions need to be transparent and clearly show that these principles are incorporated into their syllabus and daily practices. However, as pointed out by the participants, several challenges may hinder this practice—poor knowledge and understanding, lack of education, loss of ’horsemanship’ skills, commercial pressures, and pressure for success—all of which compromise correct training.

### 4.4. Cultural Change

Whilst it was widely accepted that an ethical training system incorporating these scientific principles is essential to meet equine welfare needs, a broader cultural change is necessary to ensure their acceptance and implementation across the entire equine industry. Failure to achieve this jeopardizes the SLO. Leading equestrian education institutions, NGB, FEI, and IGEQ, along with other stakeholders, have the potential to drive and shape the cultural changes needed to improve horse welfare outcomes.

Wolframm and colleagues [13] highlight that cultural change can be achieved through the incorporation of effective human behavior change interventions. In this way, a strategy for enhancing equine welfare can be developed, helping to secure the future of equestrianism.

Human behavior change is a multifaceted process involving the alteration of actions, habits, or attitudes within individuals [28]. This phenomenon is pivotal across various domains including health, environment, and social interactions. Understanding the mechanisms underlying behavior change is essential for fields such as equestrian education. Several theoretical frameworks have been proposed to elucidate this process. Among these, the Transtheoretical Learning Model delineates stages through which individuals progress in altering behavior [29], while the Health Belief Model emphasizes the role of beliefs in influencing behavior change [30]. Social Cognitive Theory underscores the impact of social influences and self-efficacy [31,32], while the Theory of Planned Behavior focuses on attitudes, norms, and perceived behavioral control [33]. The Social Ecological Model highlights that individual behavior is influenced by multiple levels, including individual, relational, organizational, community, and policy levels. The model advocates for interventions that integrate multiple levels simultaneously to achieve greater effectiveness [34], and Self-Determination Theory emphasizes intrinsic motivation [35]. Furthermore, Behavioral Economics integrates psychological and economic insights to understand decision-making and behavior change, emphasizing nudges and incentives [36].

The COM-B model, which is part of the broader Behavior Change Wheel framework, offers a systematic approach to understanding and promoting behavior change [37]. COM-B consists of three components: capability, opportunity, and motivation. Capability refers to an individual’s psychological and physical capacity to engage in a behavior, encompassing knowledge, skills, and abilities. Opportunity comprises external factors that facilitate or hinder behavior, including environmental and social influences. Motivation encompasses psychological processes that energize and direct behavior, incorporating both automatic and reflective processes influenced by intrinsic and extrinsic factors. Wolframm et al. [13] show that the use of the COM-B model, combined with coordinated efforts from multiple stakeholders to achieve behavior change, can provide the equestrian sector with valuable tools for the practical implementation of a future welfare strategy aimed at safeguarding the sport’s future. Interventions that use the COM-B model target one or more of these components based on an analysis of the specific behavior and context. By addressing capability, opportunity, and motivation, interventions aim to effectively promote behavior change across diverse settings and populations, offering a comprehensive approach to understanding and influencing human behavior.

To change a culture and create awareness of taken-for-granted norms and values in equestrian sport, a systematic and strategic approach is required, one that allows for self-reflection, questioning of existing beliefs, and the development of critical thinking [38]. The COM-B model [37] shows that the focus should not only be on transferring knowledge and skills to increase awareness of equine welfare but also on how educators can change and develop students’ mindsets, attitudes, and patterns of behavior. The COM-B model aims to create a deeper understanding and awareness of the factors that influence behavior, specifically focusing on the components of Capability, Opportunity, and Motivation, which together determine whether a person will engage in a particular behavior. By understanding these factors, interventions can be designed to promote behavior change effectively and transform it into action. As Human Behavior Change is a new topic of research in equestrianism, further research is needed to investigate which model is the most effective in generating change in this industry, as models may differ in various equestrian contexts.

### 4.5. Education: A Pedagogical Approach to Developing Students’ Ethical Mindset

Education is the driving force for change, but one of the issues voiced by the equestrian educators in this study was the challenge of teaching ’ethical training principles’ to Generation Z. This may be due to a change in behavior, but a reconsideration of the pedagogical approach is needed. Ethics and morality are often treated as synonymous. Aristotle stated, ‘Educating the mind without educating the heart is no education at all’. We all have moral beliefs and values that express our views about character and conduct: what is the right sort of person to be and what is the right thing to do. There are aspects of pedagogy used to develop this, such as challenge, explanation, modeling, practice, feedback, and questioning [39]. Teaching equestrian professionals to integrate ethical principles into their practices is essential. One approach is to incorporate ethical education into equestrian training programs, equipping professionals with the knowledge and skills needed to navigate ethical dilemmas effectively. Additionally, mentorship and role modeling by experienced equestrians can serve as powerful tools for passing on ethical values to novice equestrians [40]. Including case studies and ethical decision-making frameworks in the curriculum could help develop critical thinking and ethical reasoning skills [41]. Furthermore, ongoing professional development opportunities and reflective practices would enable coaches to continuously refine the ethics of their coaching [42]. By incorporating education on ethics, educators can provide students with the opportunity to generate knowledge about ethics and promote moral development among riders.

### 4.6. Communication with the Public

Academics and equestrian educators can disseminate research on equine welfare to the public by utilizing various channels such as scientific publications, media interviews, and public talks [43]. Presenting findings and theories in accessible language and engaging formats, such as infographics or videos, can enhance understanding [44]. Collaborating with journalists and leveraging social media platforms can broaden reach [45]. Emphasizing positive outcomes and practical solutions fosters a constructive narrative. By prioritizing transparency, credibility, and relevance, academics can effectively communicate the importance of equine welfare to diverse audiences, ultimately contributing to informed decision-making and positive societal change.

Pearson et al. [12] argue that public acceptance of equestrian sports largely depends on how the equestrian industry manages its reputation. Instead of becoming defensive or dismissing criticism, it is better to acknowledge the concerns raised, recognizing them as relevant and important. Based on Pearson et al.’s [12] study, educators can focus on several key points to prevent issues and communicate how they ensure equine welfare. These include prioritizing equine welfare issues, highlighting good examples of equine management to disseminate knowledge of best practices, consulting researchers on what constitutes good equine welfare, and adopting an open-minded, humble, curious, transparent, non-judgmental, and non-confrontational approach. Finally, educators should work with other branches of the equestrian industry to increase social acceptance.

## 5. Limitations

The World Café approach [19], a dynamic method utilized in focus group research, offered several advantages, including its ability to foster open dialogue, elicit diverse perspectives, and promote collaboration among participants. However, despite its merits, this approach is not without limitations. One such limitation is the potential for discussions to lack depth due to time constraints and the rotating nature of conversations. While the moderators’ role was to balance discussions, there was a risk of certain individuals dominating the discourse, which could limit the diversity of viewpoints represented. Moreover, maintaining focus on the main research objectives amidst multiple simultaneous conversations can be challenging. Additionally, the composition of participants may not always be representative of the broader population, which could limit the generalizability of the findings. Logistical challenges, such as coordinating equal participation and synthesizing diverse insights, also present obstacles. The potential for social desirability bias among participants may compromise the validity of the research findings. While English was the language used, it was not the first language for all participants, which may have influenced confidence, expression, meaning, and phrasing. Lastly, a definition for ’ethics’, ’ethical training’, or ’equine welfare’ was not established at the beginning of the workshop. Opinions on what is ’ethical’ and ’ethical training’ may differ among the participants. This is a complex area, and clarification among equine professionals is needed. There is an opportunity to investigate this through future research and provide recommendations and learning opportunities within the industry.

## 6. Conclusions

The aim of this study was to highlight and summarize the experiences and perceptions of European equestrian educational experts from a workshop focused on educating current and future professionals. Together, they have investigated the attitudes, views, and challenges faced by the leading European equestrian education institutions, particularly in light of the threats to equestrianism’s Social License to Operate (SLO). The results revealed that recent reports of abusive training practices are threatening the SLO, and that key stakeholders are deeply concerned that the equestrian industry is at a critical tipping point. A universal cultural change is needed, one that incorporates a clear, evidence-based approach to riding and training horses, with equine welfare at its core. The findings of this study suggest that social acceptance can only be achieved if people understand, know, and apply evidence-based practices. Current research highlights the importance of understanding and effectively applying the Five Domains model [2] and the First Training Principles [27].

Challenges such as anthropomorphism, lack of knowledge, and the changing relationship between horses and humans, as well as differences in standards across disciplines and countries, threaten the SLO. Equestrian education institutions involved in this study, due to their shared values, are in an ideal position to influence change and champion evidence-based training practices as part of their new mandate to educate future generations of riders and coaches. Participants noted that this may present a pedagogical challenge, and more research is needed in this area.

The findings from this study also highlighted the need to communicate ‘good news stories’ that showcase evidence-based knowledge in training and competition. This should be done through a wide variety of media outlets, with particular emphasis on engaging with modern social media. It was also acknowledged that all areas of the equestrian community require a unified voice and shared purpose. This can only be achieved through partnerships, sharing best practices, and engaging in regular workshops, such as those seen in this study. Further research is needed in the field of equitation science to demonstrate the benefits of horse welfare and evidence-based training practices, and to develop effective pedagogical strategies for disseminating this research.

## Figures and Tables

**Table 1:** Qualitative content analysis of the focus groups.

Higher Order Topics	Lower Order Themes	Challenges
Horse–Human relations	Relation between horse and rider	Anthropomorphism
	Changing relationship with the horse	Acceptance and dissemination of research
	Safety for horse and rider whilst interacting	Addressing knowledge gaps to prevent and combat substandard practices
	Communication and interaction	Lack of learning theory and general horsemanship skills of people entering the industry
Shared Values	Desire for a sustainable future	Differing legislation and regulations governing equestrianism
	Recognition of the need for collaboration	Cultural differences worldwide
	Shared challenges	Differing cultural rules and regulations across the disciplines
		Participation, student enrolment numbers, timetable constraints
Training Philosophy	Ethically based	Must be evidence-based training methods
	Transparent	Application of equitation science and equine learning theory
	Consideration of the individual horse	Teaching in an effective ‘horse centered’ manner
	Awareness of norms and traditions	Constraints in educational institutions (timetabling, syllabus, student numbers, facilities, etc.)
Social Acceptance	Based on research, ‘evidence-based’	Communicate within the industry and outside
	Expert knowledge	Need pedagogical skills to teach the next generation of students
	Lead stakeholders in equestrian education—position to influence change and educate the next generation of coaches and riders.	Unethical, poor practice, and horse abuse stories
	Positive examples of good practice	Phones with camera, social media, fake news, etc.
	Requires reflective practice	Individual responsibility

## Data Availability

Data are unavailable due to privacy.
